# Evaluation of Amikacin Pharmacokinetics in Critically Ill Patients with Intra-abdominal Sepsis

**DOI:** 10.15171/apb.2020.014

**Published:** 2019-12-11

**Authors:** Bita Shahrami, Farhad Najmeddin, Mohammad Reza Rouini, Atabak Najafi, Kourosh Sadeghi, Shahideh Amini, Seyedeh Sana Khezrnia, Hamid Reza Sharifnia, Mojtaba Mojtahedzadeh

**Affiliations:** ^1^Department of Clinical Pharmacy, Tehran University of Medical Sciences, Tehran, Iran.; ^2^Department of Pharmaceutics, Tehran University of Medical Sciences, Tehran, Iran.; ^3^Department of Anesthesiology and Critical Care, Tehran University of Medical Sciences, Tehran, Iran.; ^4^School of Pharmacy, Tehran University of Medical Sciences, Tehran, Iran.

**Keywords:** Amikacin, Aminoglycoside, Critical Illness, Intra-abdominal Infection, Pharmacokinetics, Sepsis

## Abstract

***Purpose:*** Although the current widespread use of amikacin is in intra-abdominal sepsis treatment, its pharmacokinetic changes in the present setting are not yet well known. This study was aimed to evaluate the amikacin pharmacokinetic profile in critically ill patients with intraabdominal sepsis compared to pneumosepsis.

***Methods:*** Adult septic patients received amikacin therapy were studied. Patients with intraabdominal sepsis were enrolled in group 1 (n=16), and patients with pneumosepsis were enrolled in group 2 (n=13). The amikacin serum concentrations were evaluated in the first, second, fourth and sixth hours after initiating 30-minute infusion. The pharmacokinetic parameters were calculated for each patient.

***Results:*** There was no significant difference in the volume of distribution between the two groups (0.33±0.08 vs. 0.28±0.10 L/kg, *P*=0.193). The amikacin clearance was significantly lower in group 1 compared to group 2 (58.5±21.7 vs. 83.9±37.0 mL/min, *P*=0.029). There was no significant correlation between amikacin clearance and creatinine clearance estimated by Cockcroft-Gault formula in all patients (*P*=0.206). The half-life was significantly longer in group 1 compared to group 2 (5.3±2.8 vs. 3.4±3.2 hours, *P*=0.015).

***Conclusion:*** Pathophysiologic changes following intra-abdominal sepsis can affect amikacin pharmacokinetics behavior. The clearance and half-life may change, but the alteration of the volume of distribution is not significantly different in comparison with pneumosepsis. Further studies are required to evaluate the pharmacokinetic variables of amikacin in critically ill patients with intra-abdominal sepsis.

## Introduction

Intra-abdominal infections (IAIs) are sepsis common causes in critically ill patients associated with a high rate of mortality and morbidity.^1^ According to IAI guidelines, one of the key strategies in the successful management of these infections is early initiation of optimal antimicrobial therapy.^2-4^ Complicated IAIs often have polymicrobial nature and require a parenteral antibiotics combination for the treatment. Therefore, it is necessary to select an appropriate antimicrobial agent with individualized dosage.^5,6^ In cases suspected with IAI aminoglycoside therapy in combination with a beta-lactam is recommended once a day, due to multi-drug resistance gram-negative organisms. Due to the increased prevalence of mentioned microorganisms, this recommendation has been more pronounced recently.^7^

Among aminoglycosides, amikacin has a valuable role in treating septic patients, as a preferable agent in intensive care units (ICUs). Like other aminoglycosides, amikacin has concentration-dependent anti-bactericidal activity with a post-antibiotic effect against certain gram-negative bacteria.^8^ Although the widespread use of amikacin in critical illnesses in recent decades, likewise the optimal dosage of this antibiotic is controversial. Conventional dosage strategies obtained from well-controlled studies are unlikely to achieve adequate drug serum concentration in non-critically ill patients.^9,10^ Indeed, critically ill patients have certain conditions causing significant changes in the pharmacokinetic profile of many drugs, particularly antibiotics, due to the pathophysiological complexity. It may lead to unpredictable serum levels and failure in treatment.^11,12^ Therefore, individualized dosage, less toxicity, and better outcomes may be achieved by considering these alterations.

The pharmacokinetics of amikacin in critically ill patients has been investigated in several studies. In this population, as a result of capillary leak, multi-organ dysfunction, and significant pathophysiological changes, such as increased cardiac output, augmented renal clearance, and hypoalbuminemia, amikacin pharmacokinetic changes include an increase in both of the volume of distribution and drug clearance leading to prolonged half-life.^13-15^ These changes may lead to sub-therapeutic drug level and poor control of infection.^16-19^ Pathophysiological changes following abdominal involvement in sepsis may affect the pharmacokinetic profile of drugs different from other forms of sepsis. Also, fluid shifting from the intravascular compartment into the interstitial or large third spacing, administrating massive volume of resuscitation fluid, multiple laparotomies, and surgeries lead to significant local extravascular fluid accumulation in the abdominal cavity. In addition, perfusion impairment and organ dysfunction may occur as a result of intra-abdominal hypertension. All of the items mentioned above can affect the volume of distribution, drug clearance, and hydrophilic antibiotics half-life.^20-22^

Despite the long history of aminoglycosides administration in septic patients with the abdominal origin, very few trials have evaluated the adequate descriptions of pharmacokinetic behavior and alteration of the volume of distribution of these agents in this population. The aim of the present study was to evaluate the amikacin pharmacokinetic profile in critically ill patients with intra-abdominal sepsis compared to pneumosepsis.

## Materials and Methods

This prospective and single-center study was performed in the general and emergency ICUs of Sina hospital affiliated to Tehran University of Medical Sciences (TUMS), Tehran, Iran. Adult critically ill patients included in this study met the criteria for sepsis or septic shock adopted by Surviving Sepsis Campaign guidelines.^23^ The patients were assigned to one of the two following groups: Patients with sepsis due to IAI were enrolled in group 1, and patients with sepsis from pneumonia were enrolled in group 2. The clinical criteria for IAI included the presence of fever or leukocytosis and at least two of the following signs and symptoms: localized or diffuse abdominal wall rigidity and/or involuntary guarding; abdominal tenderness or pain; nausea or vomiting, and/or ileus or hypoactive bowel sounds; any highly suggestive imaging findings of IAIs, such as perforation abscess, etc. Pneumosepsis is defined as nosocomial pneumonia developing 48 hours or longer after admission in ICU. Patients with severe renal impairment requiring dialysis, severe hepatic impairment and with any amikacin contraindications were excluded from the study.

Patients in both groups received 15−20 or 20−25 mg/kg empirical dose of amikacin through a 30-minute intravenous (IV) infusion based on hospital protocols on IAIs or pneumonia respectively. The patients also received other standard antimicrobial therapies and ICU care, based on staff decisions. Amikacin serum concentrations were obtained from the central venous line in the 1st, 2nd, 4th, and 6th hours after the initiating IV infusion. 2 mL of blood was drawn and dropped out after flushing the line with 5 mL of normal saline. Every sample was centrifuged at room temperature with 3000 rpm, and the serum was stored at –70°C freezer until analysis. The amikacin serum concentrations were determined using Fluorescence Polarization Immunoassays kit and Roche Diagnostics Gmbh (Mannheim, Germany).

The following amikacin pharmacokinetic parameters were calculated for each patient using one-compartment distribution model: the elimination constant rate (K_el_) as the regression line slope of natural logarithm concentration-time curve; the half-life (T_1/2_) which calculated as T_1/2_ = 0.693 / K_el_; the volume of distribution (V_d_) based on concentration at the time zero with the individual concentration-time curve to time zero extrapolation; and the amikacin clearance (CL_amk_) calculated as CL_amk_ = K_el_ × V_d_. All relevant demographic data (e.g. age, gender, and body weight), laboratory and biochemistry tests (e.g. complete blood count, electrolytes, urea, serum creatinine, and liver function tests), and sequential organ failure assessment (SOFA) scores were recorded for all patients. Creatinine clearance was estimated using the Cockcroft-Gault formula.^24^

All collected data were analyzed using SPSS software version 25. Descriptive-statistics investigation was performed on all variables. After checking normality with the Shapiro-Wilk test, Pearson correlation test and independent sample t-test were used for evaluating correlations and comparisons between averages of the two studied groups respectively. For all tests, *P*value <0.05 was statistically considered significant.

## Results and Discussion

Among thirty-seven ICU patients met the study criteria, eight patients were excluded: (i) Three patients died because of necrotizing pancreatitis, gangrenous appendicitis, and acute respiratory distress syndrome, (ii) two patients had implausible pharmacokinetics conditions, (iii) one patient developed acute renal failure, (iv) one patient discharged earlier from ICU, (v) in one patient amikacin was stopped due to an aminoglycoside resistant organism found in his blood culture. Finally, twenty-nine critically ill patients (20 men and 9 women) were enrolled with an average age of 56.6 years ranging from 30 to 80 years. 16 patients were enrolled in group 1 and 13 in group 2.

**Figure 1:**
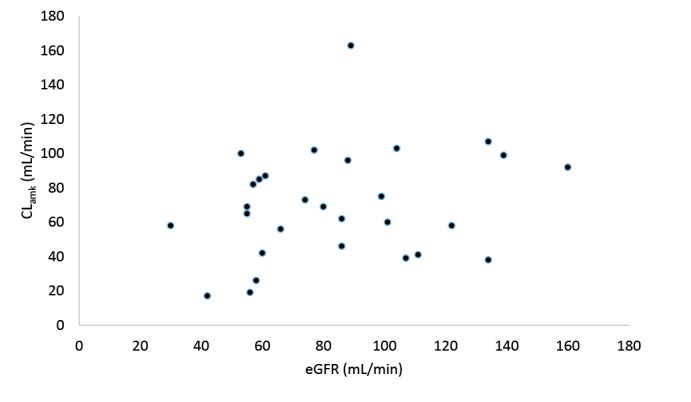
Correlation between amikacin clearance (CL_amk_) and estimated creatinine clearance (eGFR) in the patients.

As shown in Table 1, two groups were similar in terms of demographics (including gender, age, weight, and height) and SOFA scores. But the baseline of creatinine clearance was lower in group 1 compared to group 2 (72.7 ± 26.6 vs. 98.4 ± 33.9 mL/min, *P*= 0.031). Table 2 indicates the summary of amikacin pharmacokinetic parameters in both groups. Patients in group 1 received lower doses of amikacin rather than group 2 (range: 1000−1500 mg/day vs. 1500−1750 mg/d). There was no significant difference in the volume of distribution per kilogram between the two groups (0.33 ± 0.08 vs. 0.28 ± 0.10 L/kg, *P*= 0.193). The amikacin clearance was significantly lower in group 1 compared to group 2 (58.5 ± 21.7 vs. 83.9 ± 37.0 mL/min, *P*= 0.029). The amikacin clearances in group 1 was 29.9 [5.3-54.6] mL/min lower than the group 2 (*P*= 0.019) even after adjusting the groups for creatinine clearance (Cockcroft-Gault formula) using covariance test; although the Pearson correlation test showed that there was no significant correlation between amikacin clearance and estimated creatinine clearance in the patients (*R*= 0.24, *P*= 0.206) (Figure 1). The elimination constant rate and the half-life were significantly lower and longer respectively in group 1 compared to group 2 (0.15 ± 0.06 vs. 0.27 ± 0.10 h^-1^, *P*= 0.002 and 5.3 ± 2.8 vs. 3.4 ± 3.2 h, *P*= 0.015).

**Table 1:** Demographic characteristics of patients

**Measures**	**Mean ± SD**		**Sig. (2-tailed)**
	**Group 1(n=16)**	**Group 2** **(n=13)**	
Gender (male/female)	10/6	10/3	0.404
Age (y)	57.8 ± 13.1	55.2 ± 13.5	0.608
IBW (kg)	70.6 ± 8.2	69.6 ± 3.7	0.686
SOFA score	5.5 ± 4.1	5.2 ± 1.9	0.831
eGFR (mL/min)	72.7 ± 26.6	98.4 ± 33.9	0.031

Abbreviations: SD, standard deviation; IBW, ideal body weight; SOFA, sequential organ failure assessment; eGFR, estimated glomerular filtration rate.

**Table 2:** Pharmacokinetic parameters of amikacin

**Measures**	**Mean ± SD**		**Sig. (2-tailed)**
	**Group 1(n=16)**	**Group 2** **(n=13)**	
Dose/kg (mg/kg)	16.5 ± 3.0	21.8 ± 2.2	0.000
K_el_ (h^-1^)	0.15 ± 0.06	0.27 ± 0.10	0.002
T_1/2_ (h)	5.3 ± 2.8	3.4 ± 3.2	0.015
V_d_/kg (L/kg)	0.33 ± 0.08	0.28 ± 0.10	0.193
CL_amk_ (mL/min)	58.5 ± 21.7	83.9 ± 37.0	0.029

Abbreviations: SD, standard deviation; K_el_, elimination constant rate; T_1/2_, half-life; V_d_, volume of distribution; CL_amk_, clearance of amikacin.

The abdominal cavity is the second most common site of sepsis leading to serious complications. Progression of peritonitis may lead to the development of abdominal compartment syndrome (ACS) with a high rate of mortality.^25^ On the other hand, secondary ACS may develop as a result of sepsis with no evidence of previous abdominal injury.^26^ In closing, the appropriate management of intra-abdominal sepsis is crucial to reduce fatal complications. Administration of amikacin in intra-abdominal sepsis has become increasingly widely used because of the appropriate efficacy of this agent against the invasive gram-negative pathogens as well as the lack of existing antibiotics’.^27^ Yet, optimizing dosing of amikacin in the heterogeneous population of critically ill patients is challenging.

This study may provide considerable insight into amikacin pharmacokinetics in patients with intra-abdominal sepsis. The results suggested that amikacin pharmacokinetics behavior can be affected by pathophysiological changes following abdominal involvement in sepsis. As far as we know, this is the first research evaluating the amikacin pharmacokinetics specifically in septic patients with the abdominal origin.

In all studied patients, the mean amikacin volume of distribution was greater than healthy population (0.30 vs. 0.25 L/kg), which is in good agreement with previous findings indicating that increased fluid shifts in sepsis result in a larger volume of distribution for water-soluble antibiotics.^18,28^ Also, beta-lactams pharmacokinetic studies’ findings in IAI patients highlighted that systemic third spacing led to significant changes in distribution and higher volume of distribution values of these antibiotics than the healthy population.^20,29^ The mean volume of distribution in patients with intra-abdominal sepsis was higher than pneumosepsis patients, however, it was not statistically meaningful. A study by Gous et al showed that intra-abdominal sepsis does not change the ciprofloxacin pharmacokinetic parameters in comparison with sepsis with other origins, suggesting that fluid shifts have no significant effect on the volume of distribution of this lipophilic antibiotic.^30^ As amikacin has an intravascular distribution in body, the distribution of this antibiotic may increase in any conditions with increased extracellular fluid compartment.^31,32^ Liberal versus restrictive intravenous fluid therapy for sepsis can significantly affect amikacin distribution volume. But massive resuscitation fluid in IAIs may lead to visceral edema, and free intraperitoneal fluid resulting reduction in abdominal compliance may potentially lead to an increase in the volume of distribution.^33^ Therefore, while the restricted fluid strategy is carefully managed, the volume of distribution in patients with intra-abdominal sepsis may not increase and vary as expected compared to patients with pneumosepsis.

There was no significant correlation between amikacin clearance and estimated glomerular filtration rate (eGFR) in our patients, and the amikacin clearance was found to be decreased in patients with intra-abdominal sepsis. The clearances were adjusted due to the basic creatinine clearance difference between the two groups. Even after this adjustment, the studied groups were different in terms of amikacin clearance. Amikacin is primarily eliminated by the glomerular filtration with only a small fraction undergoing tubular reabsorption.^34^ The results indicated that there was no predictive value for eGFR the amikacin clearance in patients with intra-abdominal sepsis using Cockcroft-Gault formula. The unpredictability of the GFR based on serum creatinine in these patients may be due to the variable creatinine production. In this situation, a more accurate estimation of renal function may be presented by the amikacin clearance itself. In other words, this equation should be used with caution for amikacin dose adjustment in critically ill patients with intra-abdominal and pneumonia sepsis since there was a poor correlation between amikacin clearance and estimated creatinine clearance using Cockcroft-Gault formula.

Furthermore, the elimination constant rate depends on both drug clearance and volume of distribution. The studied patients have different amikacin half-lives due to the difference in the clearances and similarity in the volume of distribution in two groups.

Amikacin pharmacokinetics alterations following critical illnesses have been evaluated in many studies. In a study by Mahmoudi et al, the mean ± SD of the volume of distribution and clearance of amikacin were reported 0.36 ± 0.07 L/kg and 3.88 ± 0.97 mL/min/kg respectively.^28^ The range of clearance and elimination constant rate of amikacin were reported 0.55-11.5 L/h and 0.04-0.66 h^-1^ by Tholl et al.^35^ Lugo et al evaluated amikacin pharmacokinetics in 30 critically ill patients and reported a significant increase of volume of distribution as 0.47 L/kg.^36^ The mean of the volume of distribution was reported 0.37 and 0.41 L/kg respectively in the similar studies by Taccone et al and Marik et al.^10,37^ The mean ± SD of the volume of distribution was reported 0.39 ± 0.04 L/kg in the study of amikacin pharmacokinetics behavior in Iranian critically ill septic patients by Sabzeghabaee et al.^38^ No study was found to evaluate amikacin pharmacokinetics specialty in intra-abdominal sepsis.

We are aware that this study has some limitations. First, since GFR is not readily available in the clinical setting, we were unable to measure actual GFR in the patients; however, it would be more accruable to measure GFR with creatinine urine excretion in the future studies. Second, it was not possible to identify amikacin concentration in intraperitoneal fluid in patients following laparoscopy or drug clearance through surgical drains. Further data collection would be needed to evaluate the amikacin penetration into infected abdominal tissues.

## Conclusion

Amikacin pharmacokinetics in critically ill patients with intra-abdominal sepsis may not be similar to patients with pneumosepsis. Pathophysiological changes following abdominal infection in sepsis can affect both clearance and half-life of amikacin. But, while restricted fluid strategy and minimum positive fluid balance are applied, the alteration of the volume of distribution in this setting is not significantly different compared to patients with pneumosepsis. Further studies are recommended to evaluate the amikacin pharmacokinetic variables in patients with intra-abdominal sepsis admitted in ICU.

## Ethical Issues

The study protocol was approved by the Institutional Ethics Committee of TUMS (IR.TUMS.VCR.REC.1397.227).

## Conflict of Interest

Authors declare no conflict of interest in this study.

## Acknowledgments

We would like to thank all the patients participated in this study.

## References

[1] Sartelli M, Abu-Zidan FM, Catena F, Griffiths EA, Di Saverio S, Coimbra R (2015). Global validation of the WSES Sepsis Severity Score for patients with complicated intra-abdominal infections: a prospective multicentre study (WISS Study). World J Emerg Surg.

[2] Sartelli M, Chichom-Mefire A, Labricciosa FM, Hardcastle T, Abu-Zidan FM, Adesunkanmi AK (2017). The management of intra-abdominal infections from a global perspective: 2017 WSES guidelines for management of intra-abdominal infections. World J Emerg Surg.

[3] Mazuski JE, Tessier JM, May AK, Sawyer RG, Nadler EP, Rosengart MR (2017). The surgical infection society revised guidelines on the management of intra-abdominal infection. Surg Infect (Larchmt).

[4] Solomkin JS, Mazuski JE, Bradley JS, Rodvold KA, Goldstein EJ, Baron EJ (2010). Diagnosis and management of complicated intra-abdominal infection in adults and children: guidelines by the Surgical Infection Society and the Infectious Diseases Society of America. Clin Infect Dis.

[5] Swenson BR, Metzger R, Hedrick TL, McElearney ST, Evans HL, Smith RL (2009). Choosing antibiotics for intra-abdominal infections: what do we mean by “high risk”?. Surg Infect (Larchmt).

[6] Mazuski JE (2007). Antimicrobial treatment for intra-abdominal infections. Expert Opin Pharmacother.

[7] Lee YR, McMahan D, McCall C, Perry GK (2015). Complicated intra-abdominal infections: the old antimicrobials and the new players. Drugs.

[8] Begg EJ, Barclay ML (1995). Aminoglycosides--50 years on. Br J Clin Pharmacol.

[9] Gálvez R, Luengo C, Cornejo R, Kosche J, Romero C, Tobar E (2011). Higher than recommended amikacin loading doses achieve pharmacokinetic targets without associated toxicity. Int J Antimicrob Agents.

[10] Taccone FS, Laterre PF, Spapen H, Dugernier T, Delattre I, Layeux B (2010). Revisiting the loading dose of amikacin for patients with severe sepsis and septic shock. Crit Care.

[11] Boucher BA, Wood GC, Swanson JM (2006). Pharmacokinetic changes in critical illness. Crit Care Clin.

[12] Shah S, Barton G, Fischer A (2015). Pharmacokinetic considerations and dosing strategies of antibiotics in the critically ill patient. J Intensive Care Soc.

[13] van Dalen R, Vree TB (1990). Pharmacokinetics of antibiotics in critically ill patients. Intensive Care Med.

[14] Sadeghi K, Hamishehkar H, Najmeddin F, Ahmadi A, Hazrati E, Honarmand H (2018). High-dose amikacin for achieving serum target levels in critically ill elderly patients. Infect Drug Resist.

[15] Najmeddin F, Ahmadi A, Mahmoudi L, Sadeghi K, Khalili H, Ahmadvand A (2014). Administration of higher doses of amikacin in early stages of sepsis in critically ill patients. Acta Med Iran.

[16] Roberts JA, Lipman J (2006). Antibacterial dosing in intensive care: pharmacokinetics, degree of disease and pharmacodynamics of sepsis. Clin Pharmacokinet.

[17] Power BM, Forbes AM, van Heerden PV, Ilett KF (1998). Pharmacokinetics of drugs used in critically ill adults. Clin Pharmacokinet.

[18] Marsot A, Guilhaumou R, Riff C, Blin O (2017). Amikacin in critically ill patients: a review of population pharmacokinetic studies. Clin Pharmacokinet.

[19] Najmeddin F, Shahrami B, Azadbakht S, Dianatkhah M, Rouini MR, Najafi A, et al. Evaluation of epithelial lining fluid concentration of amikacin in critically ill patients with ventilator-associated pneumonia. J Intensive Care Med 2018:885066618754784. 10.1177/0885066618754784. 29471721

[20] Adnan S, Paterson DL, Lipman J, Kumar S, Li J, Rudd M (2012). Pharmacokinetics of beta-lactam antibiotics in patients with intra-abdominal disease: a structured review. Surg Infect (Larchmt).

[21] Waibel BH, Rotondo MF (2010). Damage control in trauma and abdominal sepsis. Crit Care Med.

[22] Al-Mufarrej F, Abell LM, Chawla LS (2012). Understanding intra-abdominal hypertension: from the bench to the bedside. J Intensive Care Med.

[23] Rhodes A, Evans LE, Alhazzani W, Levy MM, Antonelli M, Ferrer R (2017). Surviving sepsis campaign: international guidelines for management of sepsis and septic shock: 2016. Intensive Care Med.

[24] Cockcroft DW, Gault MH (1976). Prediction of creatinine clearance from serum creatinine. Nephron.

[25] Muresan MG, Balmos IA, Badea I, Santini A (2018). Abdominal sepsis: an update. J Crit Care Med (Targu Mures).

[26] Ball CG, Kirkpatrick AW, McBeth P (2008). The secondary abdominal compartment syndrome: not just another post-traumatic complication. Can J Surg.

[27] Krause KM, Serio AW, Kane TR, Connolly LE (2016). Aminoglycosides: an overview. Cold Spring Harb Perspect Med.

[28] Mahmoudi L, Mohammadpour AH, Ahmadi A, Niknam R, Mojtahedzadeh M (2013). Influence of sepsis on higher daily dose of amikacin pharmacokinetics in critically ill patients. Eur Rev Med Pharmacol Sci.

[29] Chandorkar G, Xiao A, Mouksassi MS, Hershberger E, Krishna G (2015). Population pharmacokinetics of ceftolozane/tazobactam in healthy volunteers, subjects with varying degrees of renal function and patients with bacterial infections. J Clin Pharmacol.

[30] Gous A, Lipman J, Scribante J, Tshukutsoane S, Hon H, Pinder M (2005). Fluid shifts have no influence on ciprofloxacin pharmacokinetics in intensive care patients with intra-abdominal sepsis. Int J Antimicrob Agents.

[31] Marik PE (1993). Aminoglycoside volume of distribution and illness severity in critically ill septic patients. Anaesth Intensive Care.

[32] Cornwell EE 3rd, Belzberg H, Berne TV, Kern JW, Henriques G, Asensio JA (1997). Aminoglycoside levels in critically ill surgical patients: the implications of physiologic criteria of sepsis. South Med J.

[33] Malbrain ML, Peeters Y, Wise R (2016). The neglected role of abdominal compliance in organ-organ interactions. Crit Care.

[34] Clarke JT, Libke RD, Regamey C, Kirby WM (1974). Comparative pharmacokinetics of amikacin and kanamycin. Clin Pharmacol Ther.

[35] Tholl DA, Shikuma LR, Miller TQ, Woodward JM, Cerra FB, Zaske DE (1993). Physiologic response of stress and aminoglycoside clearance in critically ill patients. Crit Care Med.

[36] Lugo G, Castaneda-Hernandez G (1997). Relationship between hemodynamic and vital support measures and pharmacokinetic variability of amikacin in critically ill patients with sepsis. Crit Care Med.

[37] Marik PE, Havlik I, Monteagudo FS, Lipman J (1991). The pharmacokinetic of amikacin in critically ill adult and paediatric patients: comparison of once- versus twice-daily dosing regimens. J Antimicrob Chemother.

[38] Sabzghabaee AM, Mojtahedzadeh M, Tajerzadeh H, Asasi N, Ganji MR, Mohagheghi A (2002). Pharmacokinetic behavior of amikacin in 31 iranian critically ill septic patients. DARU Journal of Pharmaceutical Sciences.

